# Research on Damage Detection of a 3D Steel Frame Model Using Smartphones

**DOI:** 10.3390/s19030745

**Published:** 2019-02-12

**Authors:** Botao Xie, Jinke Li, Xuefeng Zhao

**Affiliations:** 1 School of Civil Engineering, Dalian University of Technology, Dalian 116024, China; botaoxie@mail.dlut.edu.cn (B.X.); jinkeli@mail.dlut.edu.cn (J.L.); 2 State Key Laboratory of Coastal and Offshore Engineering, Dalian University of Technology, Dalian 116024, China

**Keywords:** damage detection, smartphones, steel frame, shaking table tests, wavelet packet decomposition

## Abstract

Smartphones which are built into the suite of sensors, network transmission, data storage, and embedded processing capabilities provide a wide range of response measurement opportunities for structural health monitoring (SHM). The objective of this work was to evaluate and validate the use of smartphones for monitoring damage states in a three-dimensional (3D) steel frame structure subjected to shaking table earthquake excitation. The steel frame is a single-layer structure with four viscous dampers mounted at the beam-column joints to simulate different damage states at their respective locations. The structural acceleration and displacement responses of undamaged and damaged frames were obtained simultaneously by using smartphones and conventional sensors, while the collected response data were compared. Since smartphones can be used to monitor 3D acceleration in a given space and biaxial displacement in a given plane, the acceleration and displacement responses of the Y-axis of the model structure were obtained. Wavelet packet decomposition and relative wavelet entropy (RWE) were employed to analyze the acceleration data to detect damage. The results show that the acceleration responses that were monitored by the smartphones are well matched with the traditional sensors and the errors are generally within 5%. The comparison of the displacement acquired by smartphones and laser displacement sensors is basically good, and error analysis shows that smartphones with a displacement response sampling rate of 30 Hz are more suitable for monitoring structures with low natural frequencies. The damage detection using two kinds of sensors are relatively good. However, the asymmetry of the structure’s spatial stiffness will lead to greater RWE value errors being obtained from the smartphones monitoring data.

## 1. Introduction

Structural health monitoring (SHM) technology has been successfully implemented to many structural applications and could be used to understand the environmental actions, loads, and behaviors of a structure subjected to various actions through solving a reverse problem, as well as to deduce structural safety and damage information using a measured structural response [1,2,3,4,5,6]. With the development of structural health monitoring, wireless sensing technology that can wirelessly collect and transmit data has received more and more attention and will become an inevitable trend [7,8,9,10]. However, the form and configuration of conventional wireless sensors mainly include a data acquisition module, a microprocessor module, a wireless communication module, and an energy module. These sensors employ the collaborative work of each module to complete the monitoring task; however, the professional standards of these numerous modules and operations also limit the generalization of their application. For this reason, the development of smartphones with a variety of different sensors, highly integrated processors, efficient network communication technology, data storage, and a large number of users provides the possibility for the comprehensive application of such smartphones to SHM.

A milestone in the development of smartphones was the first iPhone (iPhone 2G) launched by Apple in 2007 [11]. Since then, with the embedment of a variety of different sensors into one single device to enable more applications, smartphones have been applied to human health monitoring [12], vehicle maintenance services [13], motion recognition [14], seismic sensing [15,16], and optical biosensing [17]. The development of smartphones also offers opportunities for SHM [18,19]. Yu and Zhao [18] proposed the concept for SHM using smartphones in civil infrastructures. Morgenthal and Höpfner [20,21] investigated the potential use of smartphones for monitoring transient displacement. They also studied the possibilities and limitations of using smartphones for measuring oscillations. Cimellaro et al. [22] developed a system for the rapid assessment of damage using smartphones and the Web to collect information on damaged houses. Sharma and Arabinda [23] presented some civil engineering projects which can use smartphones to improve accuracy and efficiency. Akinwande et al. [24] developed a system for real-time pothole detection and traffic monitoring using smartphones and the Machine learning method. Feng et al. [25] and Ozer et al. [26] developed a crowdsourcing platform for SHM and a post-event damage assessment app. Zhao et al. [27,28,29] developed acceleration monitoring software, Orion-CC, and displacement monitoring software, D-viewer, which were applied for SHM of the Xinghai Bay Cross-Sea Bridge. Peng et al. [30] developed the smartphone software E-Explorer using mobile Bluetooth communication technology to realize information delivery in emergency events such as earthquakes. Min et al. [31] developed dynamic displacement monitoring software, RINO, which shows comparable accuracy with conventional laser displacement sensor under experimentally validated data. Ozer et al. [32] used collocated smartphone cameras and accelerometers to monitor a small-scale multistory laboratory model’s displacement and acceleration responses and compared this with conventional sensors. Kong et al. [33] used smartphones to monitor two directions’ acceleration responses of a building so as to demonstrate the potential usage as a way to monitor health states of buildings. As can be seen from the aforementioned studies, the research of SHM based on smartphones has developed rapidly and received considerable attention from various countries. Nevertheless, these research results are mainly concentrated on large structures such as bridges. Research on the use of smartphones to monitor building response under extreme events such as earthquakes remains limited and is thus the focus of this study.

This paper applied smartphones and conventional sensors to monitor a three-dimensional (3D) steel frame which was subjected to earthquake excitation on a shaking table. The structural acceleration and displacement responses were monitored and compared separately. Smartphones can also be used to monitor the three-dimensional acceleration in a given space and the biaxial displacement in a given plane; therefore, the acceleration and displacement responses of the Y-axis of the steel frame were obtained. Based on wavelet packet decomposition, the acceleration data monitored by smartphones and conventional sensors when the structure is in undamaged and different damaged states were analyzed. The relative wavelet entropy (RWE) was used to evaluate the damage of the structure.

## 2. Experimental Details

### 2.1. Monitoring Software on Smartphones

The development of two smartphone apps by previous studies, namely Orion-CC [27] and D-viewer [29], were used to monitor the structural responses. These mobile apps are built for an iOS 7.0 or higher platform and are currently available for free on the iTunes Store.

Orion-CC can access the internal accelerometer and gyroscope of an Apple iPhone, which can obtain not only acceleration and angle data, however also cable force. In this work, the smartphones which were preloaded with Orion-CC were used to monitor the acceleration response of the steel frame.

D-viewer is an app for monitoring structural dynamic displacement, using the built-in camera and the aid of a laser pointer to track and recognize a moving spot in order to determine relative displacement. When we used D-viewer, it first processed the color images captured by the camera into gray images and then processed the gray images into binarization images. A black circle printed on white paper was needed for purposes of calibration. The diameter of the black circle as the calibration reference could be set to determine the ratio between the actual size and the pixel size of the black circle in advance. During the monitoring process, D-Viewer monitors the coordinates of the laser spot centroid and displays the X and Y coordinate values of the laser spot in real time while also storing the laser spot coordinate values of each frame image in real time.

### 2.2. Steel Frame Details

**Figure 1:**
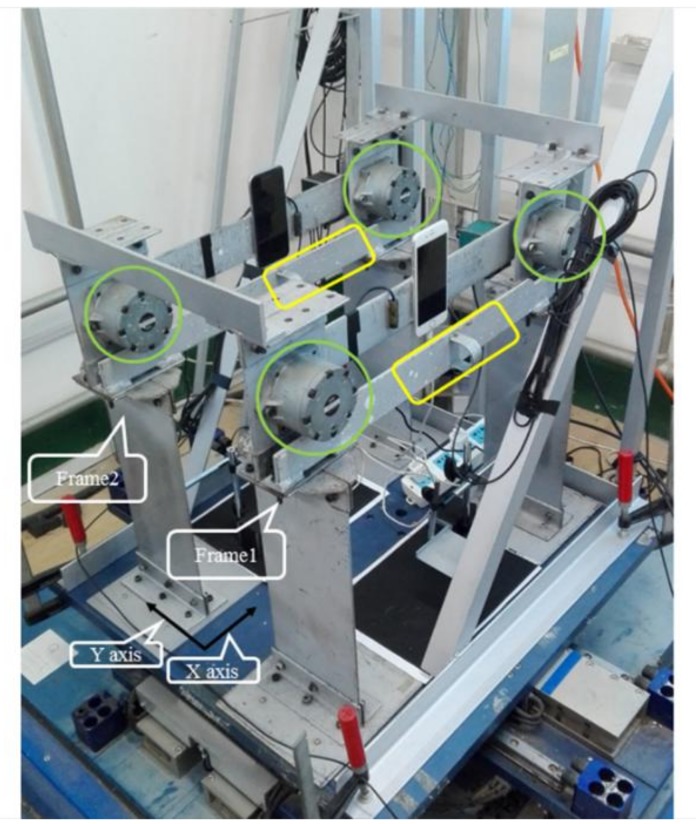
Schematic illustration of the test frame model.

The structure used in this test was a single-layer three-dimensional steel frame, which was connected by two planar steel frames (frame 1 and frame 2) through two rigid beams to form an integral frame. The experimental schematic illustration is shown in Figure 1. The frame was 500 mm in length, 400 mm in width, and 400 mm in height, and each beam-column joint was installed with a viscous damper to simulate the damage of the frame (highlighted by the green circle). A removable rigid beam was installed in front of the damper (highlighted by the yellow box). When the beams were installed, the rotary damper did not function, so the structure was in an elastic state. However, because the mass of the rigid beam is about 0.75 kg and the overall mass of the structure is about 30 kg, once the rigid beam was removed, the structural mass’ loss was small, however the stiffness was greatly reduced, the dampers were rotated, and damping of the structure could be gradually increased. Therefore, in this study, removing the rigid beam was employed to simulate the structural damage states. The basis of the structure was two rigid plates that were bolted onto a dual-axis XY Shake Table III that can support and excite loads up to 100 kg. The connection was fixed to ensure that the frame did not undergo horizontal shear translation during the vibration process.

### 2.3. Instrumentation Layout

**Figure 2:**
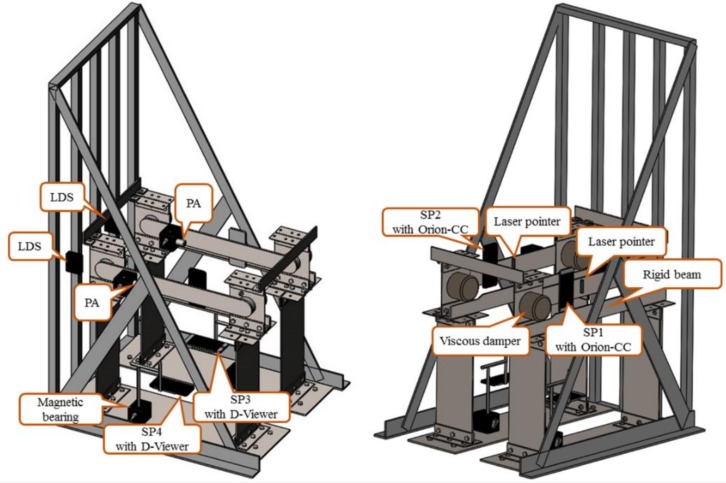
The various sensors instrumented in different locations in the steel frame testbed are shown.

The sensor device in the steel frame is shown in Figure 2. Two uniaxial piezoelectric accelerometers (PAs) were respectively mounted on the viscous dampers’ articulated beams of frame 1 and frame 2, wherein the PAs were bonded to the magnetic bearings which were instrumented on the rear plane of the articulated beams. The articulated beams were firmly connected so that the PAs and smartphones would be able to measure the acceleration responses of the test structure. The sensitivity of the accelerometer was 500 mV/g with a maximum output voltage of 6 V. In the front plane of the damper articulated beams, two smartphones (SP1 and 2) which were pre-installed with Orion-CC were attached to monitor the acceleration response of the structure and the results were compared with those of the piezoelectric accelerometers.

The triangular support frame was also attached to the shaking table so that it could move with the table. Two laser displacement sensors (LDSs) were mounted on the triangular support frame’s vertical columns and it was ensured that the outer planes of the two vertical columns were in the same planes as the two magnetic bearings on the rear plane of the damper articulated beams of frame 1 and frame 2. Then, the laser spot could be launched into the center of the magnetic bearing to monitor the relative displacement of the structure. The LDSs had a measuring range of 160 to 450 mm and an output voltage of 0 to 5V. Meanwhile, two smartphones (SP3 and 4), which were pre-installed with the D-Viewer software, were fixed at the bottom of frame 1 and frame 2, and the laser pointers were fixed on the damper articulated beams as well. When the frame began to vibrate, the laser spot that was projected on the pedestal moved laterally. Each smartphone’s camera recorded images of the laser spot and analyzed the position of the spot in each image to obtain the relative displacement of the frame. Finally, both the PAs and LDSs were connected to a data acquisition (DAQ) system.

### 2.4. Test Plan and Damage Cases

Four cases were considered in this study. The first was the undamaged case—at this point in time, the tops of the two columns of frame 1 and frame 2 were mounted with beams so that the beam-column connections were considered to be rigid and the dampers did not work. The second was the damaged 1 case, wherein the rigid beam of frame 1 was removed. The viscous dampers could be rotated to affect the structural response, and the elastoplastic behavior of frame 1 was simulated. The third was the damaged 2 case, wherein the rigid beam of frame 2 was removed and it also simulated the elastoplastic behavior of frame 2. In the last case, the rigid beams of frame 1 and frame 2 were removed simultaneously. Table 1 shows the information of all the damage cases.

**Figure 3:**
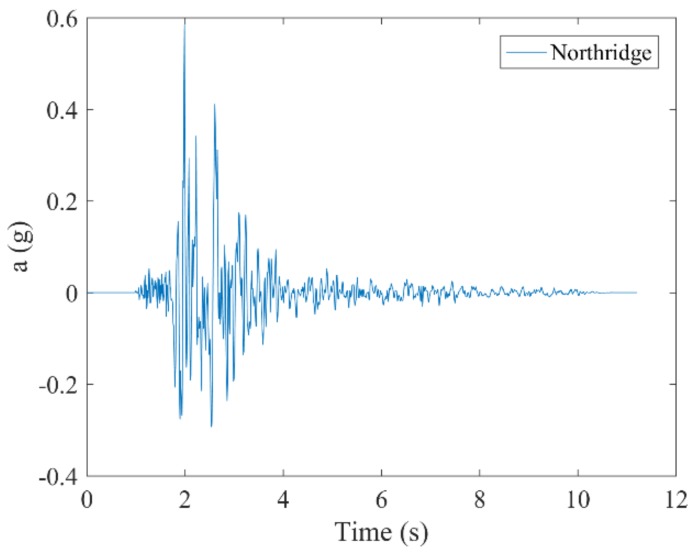
Schematic illustration of an earthquake wave.

The M_w_ 6.7 Northridge earthquake occurred on 17 January, 1994 in the San Fernando Valley, southern California and caused a large number of structural collapses and casualties [34]. Since then, many researchers have regarded this seismic wave as one of the input waves for structural seismic resistance. In this paper, the Northridge wave was employed as input, representing earthquake excitation. Since the magnitude of the input excitation was differentiated according to the peak displacement of the seismic waves, the excitation in this study was divided into two working conditions—one peak of 1 cm and another of 2 cm. The characteristics of the seismic waves are shown in Table 2, and Figure 3 shows the waveform of the seismic wave.

## 3. Steel Frame Response Comparison

### 3.1. Acceleration Time-History Comparison

**Figure 4:**
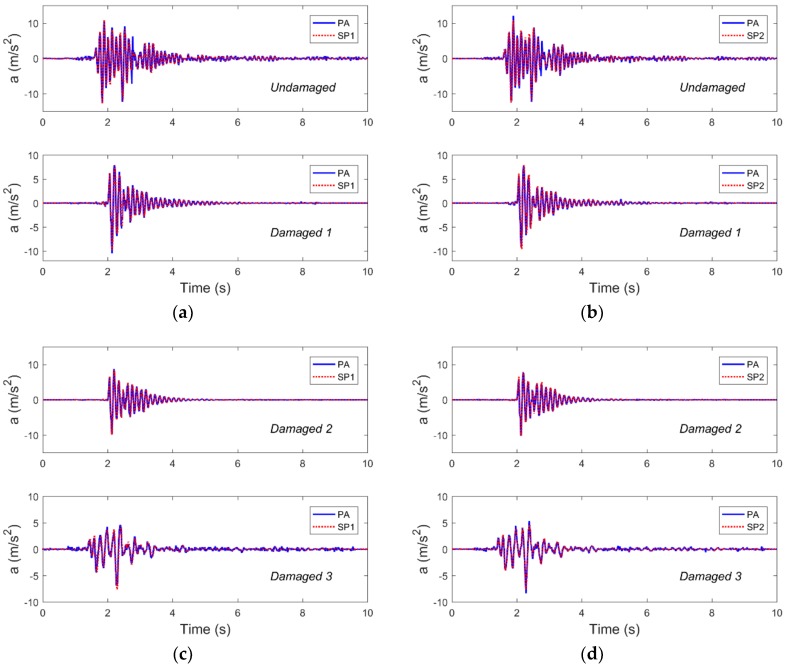
The acceleration time-history responses, as collected by the piezoelectric accelerometers (PAs) and smartphones (SPs) for four damage cases subjected to Nr-1 cm excitation, are compared. Representative results from (**a**) frame 1 and (**b**) frame 2 in the undamaged and damaged 1 cases, as well as (**c**) frame 1 and (**d**) frame 2 in the damaged 2 and damaged 3 cases are overlaid.

**Figure 5:**
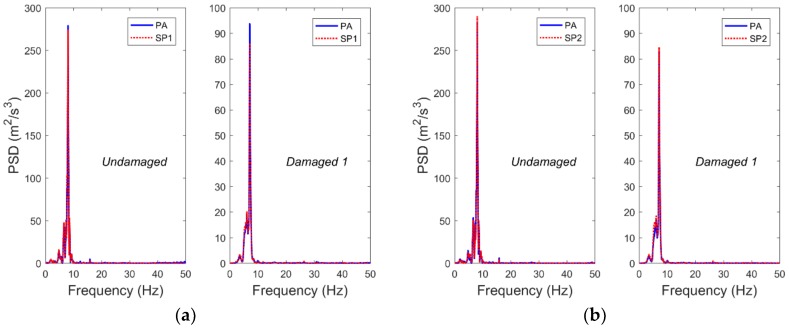

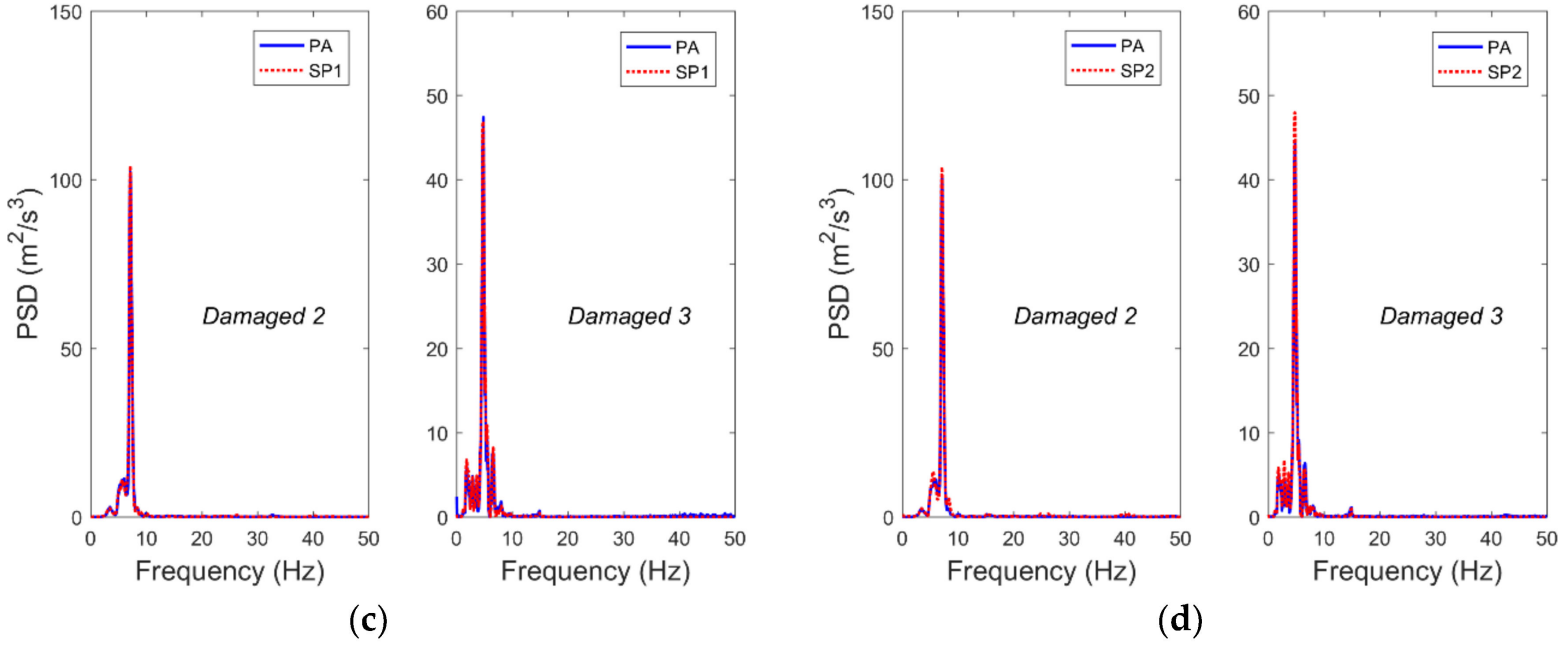
Using the acceleration time-history measurements from Figure 4, the power spectral density functions of (**a**) frame 1 and (**b**) frame 2 in the undamaged and damaged 1 cases, as well as (**c**) frame 1 and (**d**) frame 2 in the damaged 2 and damaged 3 cases are compared.

In this study, the PAs and the SPs were used to monitor the top acceleration responses of frame 1 and frame 2, and the monitoring results were compared. Due to space limitations, Figure 4 shows the results of the comparative analysis, which were used in the acceleration data of frame 1 and 2 in the different damage cases under the excitation of Nr-1 cm. As can be seen in Figure 4, the acceleration data obtained by the SPs and the PAs are well matched with each other. At the same time, by performing fast Fourier transform (FFT) on the time domain data, the power spectral density (PSD) function corresponding to the case shown in Figure 4 was obtained, as shown in Figure 5. It can be seen that the frequency domain results also have a good match. These results show that when the structure is subjected to extreme events such as earthquakes, the structure can be monitored for vibration using smartphones.

Table 3 shows the acceleration response peaks of the structures under different damage conditions monitored by the two sensors under different excitation. It can be seen from Table 3 that although some numerical anomalies are present, the acceleration peak errors monitored by the two sensors are generally within 5%. This further illustrates the reliability of vibration monitoring using smartphones.

The peak-picking algorithm was used to process the structural acceleration response data under different damage cases, and the basic modal frequency of the structure was obtained. Table 4 shows the results of the analysis. It can be seen from Table 4 that the first modal frequency of the structures in the different damage cases recognized by the two kinds of sensors are substantially the same. Meanwhile, as the damage increases, the first modal frequency of the structure gradually decreases, which is reasonable because the removed rigid beam at the corresponding location reduces the overall stiffness of the structure.

**Figure 6:**
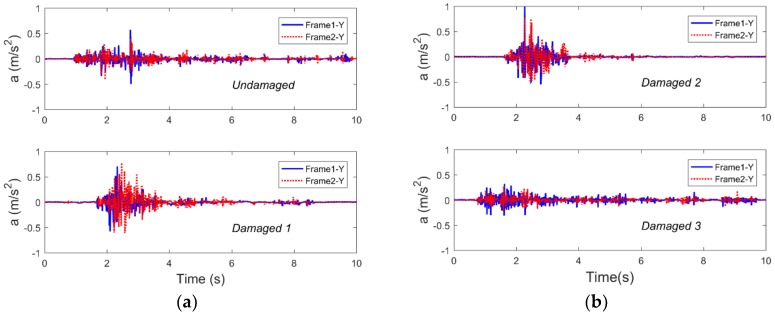
The Y-axis acceleration time-history responses as collected by smartphones subjected to Nr-1 cm are shown (**a**) in the undamaged and damaged 1 cases and (**b**) in the damaged 2 and 3 cases.

In this study, although the direction of the seismic excitation only occurred in the plane X direction, smartphones could simultaneously monitor the structural acceleration responses in the plane Y direction. Figure 6 shows the Y-direction acceleration responses of the structure in the different damage cases.

It can be seen that although the amplitude is small, the vibration trend and amplitude of frames 1 and 2 in the Y direction are similar, which may be because the columns of the model were formed by 4-mm-thick, 150-mm-wide, and 400-mm-tall steel plates. Such a column has a small outer plane (X-direction) stiffness and a large in-plane (Y-direction) stiffness, which limits the vibration and displacement of the structure in the Y direction. Despite all this, smartphones can monitor the acceleration response in the Y direction of the structure. Compared with the traditional triaxial acceleration sensors, smartphones not only can simultaneously monitor the acceleration responses in three directions, however they are also cheaper and more popular than the sensors.

### 3.2. Displacement Time-History Curves Comparison

**Figure 7:**
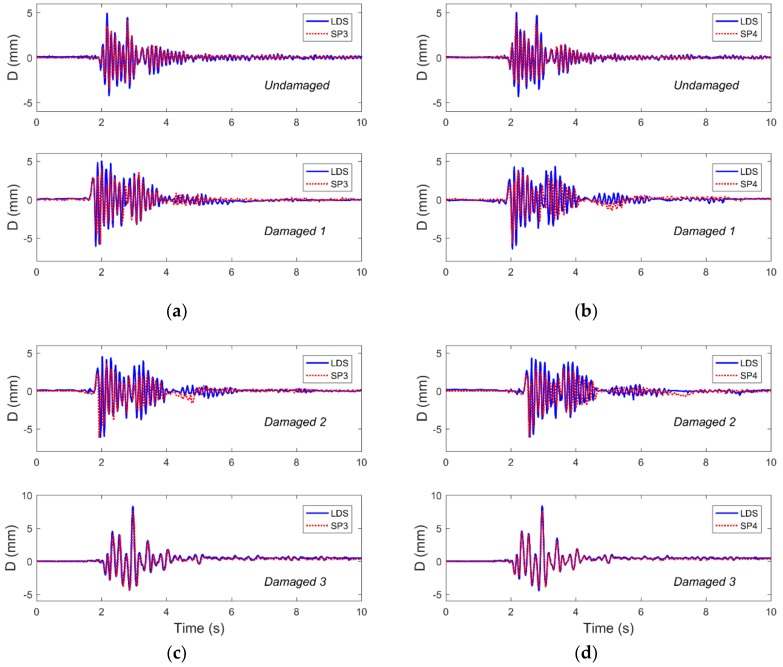
The displacement time-history responses as collected by the LDSs (laser displacement sensors) and SPs with Nr-1 cm excitation for four damage cases are compared and found to show good agreement. Representative results are shown from (**a**) frame 1 and (**b**) frame 2 in the undamaged case and damaged 1 case, as well as from (**c**) frame 1 and (**d**) frame 2 in the damaged 2 and 3 cases.

According to Section 2.1, the relative displacement of the vertex was obtained using a video image processing method recorded by smartphones, and the reference displacement measurement was collected using the LDS mounted on the top side of the structure. Also limited by space, Figure 7 shows the displacement comparison results for the representative structures monitored by the two kinds of sensors in the four cases subjected to Nr-1 cm excitation. It can be seen from Figure 7 that the displacement time-history data monitored by the smartphones are basically the same as that of the LDSs. Furthermore, compared with the case of structural damage, the data monitored by the two kinds of sensors differ greatly in peak value in the case where the structure was not damaged. This could be due to two reasons. The first reason is the effect of the sampling rate, where the sampling rate of the LDS is 100 Hz, while the sampling rate of the smartphones equipped with the D-Viewer app is 30 Hz, and the difference in sampling rate may cause measurement errors. The second reason may be that the smartphones are subject to ambient illumination, affecting the target reference (i.e., laser spot) and installation methods when recording and processing video images, thus resulting in measurement errors.

In order to analyze the difference in displacement data monitored by the two kinds of sensors, Table 5 shows the displacement response peaks of the structures with different damage cases subjected to different excitation levels. It can be seen from the table that the displacement data monitored by smartphones are more error-prone than the data monitored by LDSs in the undamaged case. This may be because the smartphone’s sampling rate is only 30 Hz. As can be seen from Table 4 above, the first model frequency of the undamaged structure is 8.05 Hz. Considering the influence of higher order frequencies on the structural displacement response, the sampling rate of 30 Hz may not satisfy the sampling theorem and results in the data’s error. When the structure is damaged, the natural frequency of the structure is gradually reduced, and the sampling rate of 30 Hz gradually satisfies the sampling demand so that the error of the monitoring result is gradually reduced. In summary, although the comparison results have some errors, they still verified the use of smartphones for displacement monitoring and indicated that smartphones could be used to monitor the interlayer displacement of structures. Compared with traditional sensors, smartphones with a displacement response sampling rate of 30 Hz are more suitable for monitoring structures with low natural frequencies.

### 3.3. Y-Axis Displacement Time-History Curves of the Steel Frame

**Figure 8:**
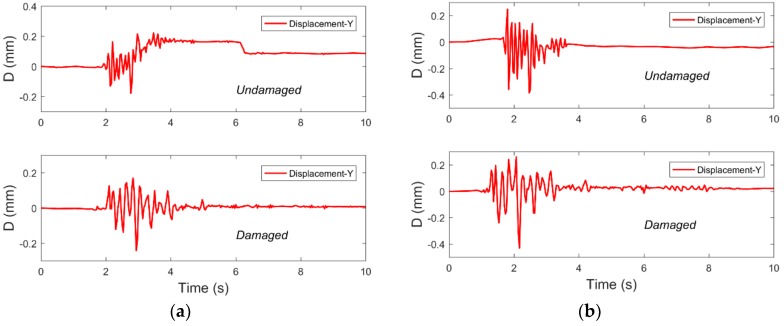
Displacement of the frame in the Y direction for (**a**) the Northridge-1 cm case and (**b**) the Northridge-2 cm case.

The D-Viewer software could measure the displacement not only in the X direction of the structure, however also in the Y direction, so that the torsional response of the structure during the vibration process could be analyzed. Due to the small displacement of the structure in the Y direction and the influence of factors such as the environment on the smartphone, some signal-to-noise ratios of the displacement data were poor. However, some good data can be used to explain some problems. Figure 8 shows the displacement of the structure in the Y-axis in undamaged and damaged states.

It can be seen that although the Y-axis displacement response of the single-layer structure model is small, smartphones still could monitor the Y-axis displacement. This may be due to the small difference in stiffness between the two planar frames, resulting in a torsional effect and displacement response in the Y direction. This result shows that when the structure is under the action of extreme events such as earthquakes, the application of smartphones can simultaneously monitor the displacement response in both directions of the structure plane, thus reducing the number of sensors required.

### 3.4. Different Frequencies of LDSs Compared with SPs

**Figure 9:**
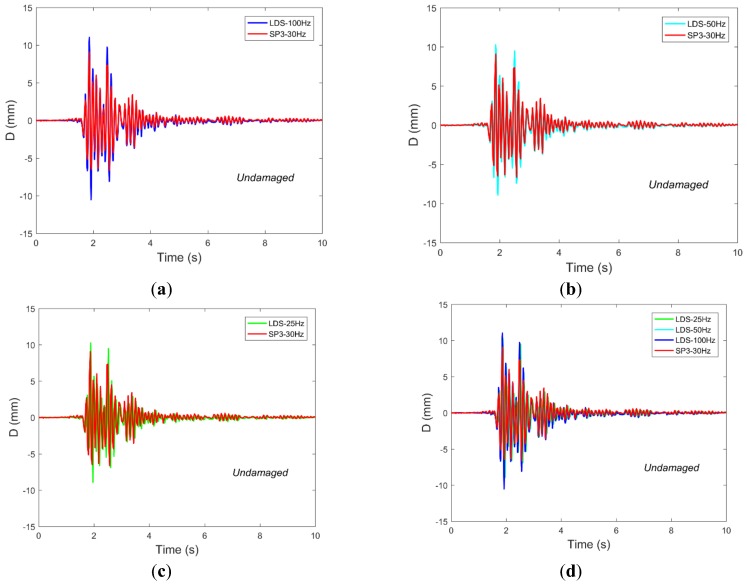
The displacement time-history responses as collected by SPs compared with LDSs using different sampling rates of (**a**) 100 Hz, (**b**) 50 Hz, and (**c**) 25 Hz. (**d**) A comparison of all sampling rates.

In order to verify the influence of different sampling rates on the displacement data, the data recorded by the LDSs were resampled at 100 Hz, 50 Hz, and 25 Hz. Compared with the displacement data recorded by the smartphones, the results are shown in Figure 9. It can be seen that in the undamaged case, the data collected by smartphones are closest to the data recorded by the LDS at a sampling rate of 25 Hz. At the same time, in the damaged case, the changes in the sampling rate of the LDS are not significantly different from the data recorded by the smartphones. This shows that the difference in sampling rate has a greater influence on structures that have a higher fundamental mode frequency. The data in Table 6 further illustrates this conclusion.

## 4. Damage Detection Results and Discussion

### 4.1. Wavelet Packet Analysis Background

Wavelet packet analysis (WPA) is a method of vibration signal processing proposed by Wu and Du in 1996 [35]. With the development of WPA, structural damage identification based on the energy of wavelet packet nodes of structural dynamic response has been widely studied [36,37,38].

According to Parseval’s theorem, the energy in the time-domain is equal to that of the frequency-domain. When damage occurs, the energy corresponding to each frequency is redistributed and the structural response of each frequency band changes.

In order to extract structural damage information from structural response signal, here we suppose that a signal, *S(t)*, can be expressed by wavelet packet decomposition:

(1) $$S(t)=\sum_{j=1}^{2^{k-1}}S_{kj}(t)$$

where *S_kj_(t)* is the sub-signal with an orthogonal frequency band and *k* indicates the layer number of the tree structure of wavelet decomposition.

The energy of these sub-signals *E_j_* can be expressed as:‖

(2) $$E_{j}=||S_{kj}(t)|{|}^{2}=\sum_{k}|S_{kj}(t){|}^{2}$$

In consequence, the total energy *E_tol_* can be obtained by:

(3) $$E_{tol}=||S(t)|{|}^{2}=\sum_{j=1}^{N}E_{j}$$

The wavelet energy ratio {*Pj*} for the *j*th scale is considered as a normalized value:

(4) $$P_{j}=\frac{Ej}{E_{tol}}$$

Clearly,

(5) $$\sum_{j=1}^{N}Pj=1$$

In this study, a damage index *S_WT_* based on relative wavelet entropy (RWE) was employed and is formulated as follows [36]:

(6) $$S_{WT}(p|q)=\sum_{j<0}p_{j}\cdot \ln[\frac{p_{j}}{q_{j}}]$$

where {*p*} is the set of damaged structural wavelet energy ratio vectors and {*q*} is the set of undamaged structural wavelet energy ratio vectors. Note that the RWE is positive and vanished only if $\left\{p_{j}\right\}=\left\{q_{j}\right\}$.

### 4.2. Wavelet Packet Decomposition of Acceleration Time-Histories

**Figure 10:**
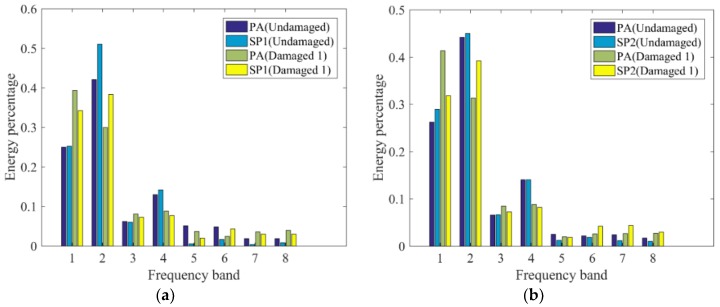

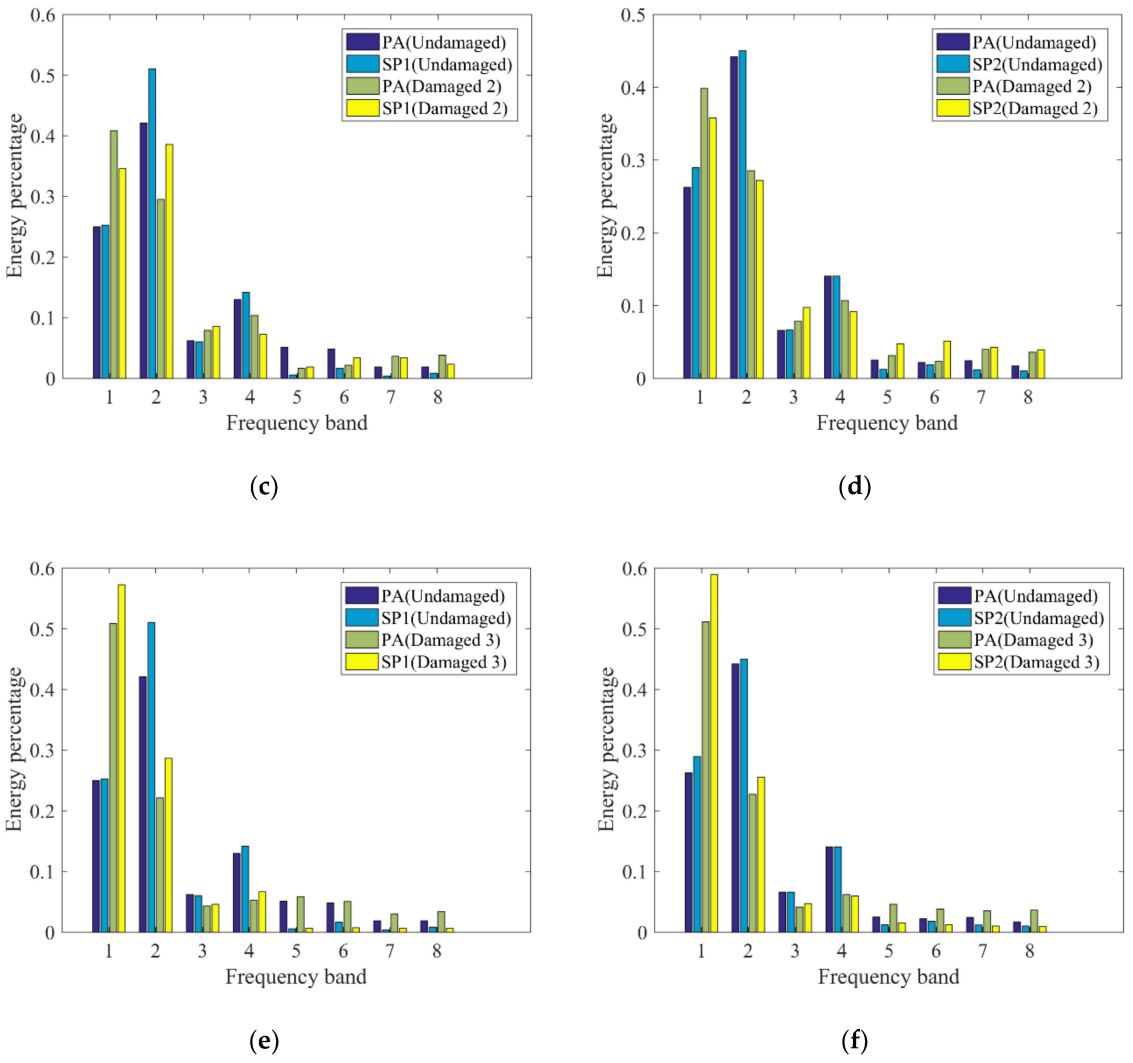
*p_j_* calculated for (**a**) frame 1 and (**b**) frame 2 in the damaged 1 case, (**c**) frame 1 and (**d**) frame 2 in the damaged 2 case, and (**e**) frame 1 and (**f**) frame 2 in the damaged 3 case when structure is subjected to Nr-1 cm excitation.

De-noising of acceleration results was achieved by decomposing the data using three-order WPA with ‘db3’ to obtain eight frequency bands. The data were decomposed into wavelet packets to obtain the energy distribution of each frequency band. *P_j_* was obtained using the acceleration data monitored by the SPs and that obtained by the PAs, as shown in Figure 10. It can be seen from the figure that the energy distribution of the three damaged systems changes at each frequency band compared with the undamaged system and is mainly concentrated on frequency bands 1 and 2. Compared with the damaged 3 system, the energy distribution change of each frequency band in the damaged 1 and damaged 2 systems are more similar. This could be caused by the same damage degree of the damaged 1 and 2 cases. Due to the rigid connection of frame 1 and frame 2, the response of the structure was forced to be similar; thus, the changes of energy distribution in each frequency band were similar. This is also consistent with the change in the fundamental mode frequency of the structure.

Table 7 shows the relative wavelet entropy coefficients of the various acceleration data for the structure with different damage cases. As can be seen from the table, for the damaged 1 and damaged 2 cases, the structure’s RWE values do not differ greatly, and the RWE of the frame from which the rigid beam was removed is larger than that of the frame with the beam. For the damaged 3 case, the structure’s RWE value is significantly increased. This indicates that the damage level of the structure was increased. This is also consistent with the change of the fundamental mode frequency of the structure. Meanwhile, the contrast of wavelet analysis results using two kinds of sensors are relatively good. For the damaged 1 and damaged 2 cases, the structure’s RWE values’ errors are larger than the damaged 3 case. This is probably because the structures of damage 1 and damage 2 only removed one rigid beam so that the structural rigidity was asymmetrical. When the structure vibrated, the eccentricity caused the acceleration responses of the two kinds of sensors attached to the two sides of the damper articulated beam to be different in different frequency bands. However, on the contrary, the structure of the damage 3 removed two rigid beams at the same time and the spatial stiffness of the structure was symmetrical, so the energy distributions of the acceleration responses monitored by the two kinds of sensors were similar in different frequency bands and the RWE values’ errors are relatively small. Despite all this, these results demonstrate the feasibility of using smartphones and corresponding damage index methods (such as RWE) for damage detection.

## 5. Conclusions

In this paper, a three-dimensional steel frame was subjected to shaking table tests simulating earthquake excitation. Three damaged cases were introduced by removing the different rigid beams in the frame to cause the viscous damper mounted at the beam-column joint to rotate. The response data of the undamaged structure and damaged structures were monitored using both conventional sensors and smartphones which were pre-installed with measurement acceleration (Orion-CC) and displacement (D-viewer) software. The data monitored by smartphones and conventional sensors were compared, and the causes of data errors were analyzed. Wavelet packet decomposition and relative wavelet entropy were employed to identify and evaluate structural damage. The conclusions are summarized as follows:

(1) The acceleration responses acquired by smartphones and piezoelectric acceleration sensors were matched quite well. In order to obtain the change of the basic modal frequency of the frame in the different damage cases, the peak-picking method was applied. The results show that the first modal frequency of the structures in the different damage cases recognized by the two kinds of sensors are substantially the same. Meanwhile, as the damage increases, the first modal frequency of the structure gradually decreases.
(2) The results of the comparison of the displacement acquired by smartphones and LDS are basically good. The influence of the sampling rate of the two kinds of sensors on the monitoring results was analyzed. The results show that compared with traditional sensors, smartphones with a displacement response sampling rate of 30 Hz are more suitable for monitoring structures with low natural frequencies.
(3) Wavelet packet analysis was used to analyze the acceleration data, and the damage index based on RWE was obtained under different damage cases. The results demonstrate that the contrast of wavelet analysis results using two kinds of sensors are relatively good. However, the asymmetry of the structure’s spatial stiffness will lead to greater RWE value errors being obtained from the smartphones monitoring data. Despite all this, the structural damage could be detected using smartphones.

In summary, this study demonstrates the feasibility of using smartphones to monitor the response of a building structure subjected to extreme events such as earthquakes. It is worth noting that because the conclusions of this paper are drawn from the model test analysis, the practical application of smartphones is not included in this paper and will be the focus of the next research. At the same time, how to improve the sample rate of the displacement collected by the smart phone through the camera should be studied.

## Figures and Tables

**Table 1:** Damage cases of the test used in this study.

Damage Case	State of the Structure
Undamaged	Rigid beams were mounted
Damaged 1	Rigid beam of frame 1 was removed
Damaged 2	Rigid beam of frame 2 was removed
Damaged 3	Rigid beams of frame 1 and frame 2 were removed simultaneously

**Table 2:** Earthquake records.

Earthquake (abbr.)	Peak Displacement (cm)	Direction of Excitation
Northridge (Nr)	1	Unidirectional
	2	Unidirectional

**Table 3:** Peak values of acceleration under different excitation levels by two kinds of sensors.

Damage Case	Earthquake Excitation	Frame 1			Frame 2		
		PA (m/s^2^)	SP (m/s^2^)	Error	PA (m/s^2^)	SP (m/s^2^)	Error
Undamaged	Nr-1 cm	12.73	12.74	0.08%	12.26	12.52	2.16%
	Nr-2 cm	27.28	28.16	3.20%	27.22	25.60	−5.98%
Damaged 1	Nr-1 cm	10.46	9.75	−6.75%	9.29	9.56	2.85%
	Nr-2 cm	18.98	19.08	0.52%	17.97	18.96	−5.51%
Damaged 2	Nr-1 cm	9.64	9.86	−2.30%	10.11	10.16	0.48%
	Nr-2 cm	19.51	20.02	−2.61%	20.26	21.28	5.03%
Damaged 3	Nr-1 cm	6.87	7.63	−11.13%	8.36	7.58	−9.38%
	Nr-2 cm	14.47	15.32	5.82%	16.50	16.53	0.18%

**Table 4:** First modal frequency results obtained using the PAs and SPs.

Damage Case	Sensor Type	Steel Frame Model	
		Frame 1 (Hz)	Frame 2 (Hz)
Undamaged	SP	8.05	8.05
	PA	8.00	8.05
Damaged 1	SP	7.05	7.10
	PA	7.05	7.05
Damaged 2	SP	7.10	7.10
	PA	7.15	7.15
Damaged 3	SP	4.80	4.75
	PA	4.85	4.80

**Table 5:** Peak values of displacement under different excitations by two kinds of sensors.

Damage Case	Earthquake Excitation	Frame 1			Frame 2		
		LDS (mm)	SP (mm)	Error	LDS (mm)	SP (mm)	Error
Undamaged	Nr-1 cm	4.931	4.194	−14.95%	5.026	4.24	−15.64%
	Nr-2 cm	11.15	9.074	−18.62%	11.7	11	−5.98%
Damaged 1	Nr-1 cm	6.066	5.763	-5.00%	6.412	5.636	−12.10%
	Nr-2 cm	13.34	10.4	-22.04%	12.48	12.23	−2.00%
Damaged 2	Nr-1 cm	6.105	6.142	0.61%	6.108	6.024	−1.38%
	Nr-2 cm	13.07	9.693	-25.84%	12.95	9.795	−24.36%
Damaged 3	Nr-1 cm	8.321	7.603	−8.63%	8.158	7.703	−5.58%
	Nr-2 cm	16.94	16.24	−4.13%	16.95	16.35	−3.54%

**Table 6:** Peak values displacement of SPs and LDSs with different frequencies.

Damage Case	Earthquake Excitation	Frame Model and Difference	SP (mm)	100 Hz (mm)	50 Hz (mm)	25 Hz (mm)
Undamaged	Nr-1 cm	Frame 1	4.194	4.931	4.549	4.489
		Error		−14.95%	−7.80%	−6.57%
		Frame 2	4.24	5.036	4.764	4.482
		Error		−15.64%	−11.00%	−5.40%
	Nr-2 cm	Frame 1	9.074	11.15	10.15	9.858
		Error		−18.62%	−18.62%	−7.95%
		Frame 2	11.00	11.70	11.70	9.81
		Error		−5.98%	-5.98	12.15%
Damaged 1	Nr-1 cm	Frame 1	5.763	6.066	6.066	5.092
		Error		−5.00%	−5.00%	13.18%
		Frame 2	5.636	6.412	6.159	6.159
		Error		−12.10%	−8.49%	-8.49%
	Nr-2 cm	Frame 1	10.4	13.34	12.82	12.17
		Error		−22.04%	−18.88%	−14.54%
		Frame 2	12.23	12.48	12.48	12.48
		Error		−2.00%	−2.00%	−2.00%
Damaged 2	Nr-1 cm	Frame 1	6.142	6.105	5.993	5.993
		Error		0.61%	2.49%	2.49%
		Frame 2	6.024	6.108	6.108	5.436
		Error		−1.38%	−1.38%	10.82%
	Nr-2 cm	Frame 1	9.693	13.07	13.07	9.265
		Error		−25.84%	−25.84%	4.62%
		Frame 2	9.795	12.95	12	10.91
		Error		−24.36%	−18.38%	−10.22%
Damaged 3	Nr-1 cm	Frame 1	7.603	8.32	8.32	8.32
		Error		−8.62%	−8.62%	−8.62%
		Frame 2	7.703	8.368	8.368	8.368
		Error		−7.95%	−7.95%	−7.95%
	Nr-2 cm	Frame 1	16.24	16.94	16.85	16.85
		Error		−4.13%	−3.62%	−3.62%
		Frame 2	16.35	16.95	16.95	16.95
		Error		−3.54%	−3.54%	−3.54%

**Table 7:** The relative wavelet entropy (RWE) index for different cases.

Damage Case	Frame Model	Earthquake Excitation	Different Sensors		
			PA	SP	Error
Damaged 1	Frame 1	Nr-1 cm	0.086	0.102	18.26%
	Frame 2		0.073	0.0577	−21.39%
	Frame 1	Nr-2 cm	0.114	0.096	−15.57%
	Frame 2		0.114	0.052	−54.00%
Damaged 2	Frame 1	Nr-1 cm	0.110	0.102	-7.61%
	Frame 2		0.079	0.136	71.59%
	Frame 1	Nr-2 cm	0.121	0.149	23.10%
	Frame 2		0.129	0.127	-0.86%
Damaged 3	Frame 1	Nr-1 cm	0.202	0.224	10.80%
	Frame 2		0.213	0.199	-6.61%
	Frame 1	Nr-2 cm	0.198	0.202	1.97%
	Frame 2		0.201	0.215	6.70%
